# Misfolding and interactions of Aß proteins: Insight from single molecule experiments and computational analyses

**DOI:** 10.1186/1750-1326-8-S1-P64

**Published:** 2013-10-04

**Authors:** Lv Zhengjian, Yuliang Zhang, Alexey Krasnoslobodsev, Robin Roychaudhuri, Margaret Condron, David Teplow, Sandor Lovas, Luda Shlyakhtenko, Yuri Lyubchenko

**Affiliations:** 1 Department of Pharmaceutical Sciences, University of Nebraska Medical Center, 986025 Nebraska Medical Center, Omaha,NE, USA; 2 Department of Neurology, David Geffen School of Medicine at UCLA, Los Angeles, USA; 3 Brain Research Institute and Molecular Biology Institute, Los Angeles, USA; 4 Department of Biomedical Sciences, Creighton University, Omaha, NE, USA

## Background

The current model for the development of Alzheimer’s (AD), Parkinson’s, Huntington’s, prion, and other neurodegenerative diseases involves protein misfolding as the early step followed by spontaneous aggregation, with specific proteins identified as the primary initiators for disease development. Therefore, elucidating the properties of the disease-prone misfolded states, understanding the mechanism of their formation, and identification of their most toxic forms will open prospects for the development of early diagnostics and specific therapeutics for these diseases.

## Materials and methods

We have developed single molecule AFM force spectroscopy (SMFS) experimental approach enabling us to probe interprotein interactions and to identify those interactions that correspond to misfolded protein states. Using SMFS, we have discovered that misfolded dimers are very stable. The following questions were addressed: *How does the misfolded dimer form? Do the monomers adopt misfolded states prior to their assembly into the dimer or the conformational transition occurs inside the dimers? What is the structure of the dimer?*

## Results

Aß42 and Aß40 are the two primary alloforms of the amyloid β-protein and we applied SMFS approaches to characterize the effects of C-terminal substitutions on the structure of transiently formed dimers. We discovered a dramatic difference in the folding patterns of Aß42 and Aß40 monomers within dimers. Although the sequence difference between the two peptides is at the C-termini, the N-terminal segment plays a key role in the peptide folding in the dimers.

To address the question on the mechanism of the misfolded dimers formation we applied Molecular Dynamics simulations. When two monomers approach, their structure changes dramatically. The arrangement of monomers in an antiparallel orientation leads to the cooperative formation of a ß-sheet conformer.

The amyloid misfolding depends on the environmental conditions and AFM is capable of characterizing these effects.

## Conclusions

Misfolding of amyloids occurs through the formation of dimers.

Misfolded dimers are conformationally stable and their formation triggers the subsequent aggregation process.

The stabilization of N-terminal interactions of Aß proteins is a switch in redirecting of amyloids from the neurotoxic aggregation pathway.

AFM is uniquely suited for developing preventions of the AD early-onset and diagnostics.

